# The Fat-Dachsous planar polarity pathway competes with hinge contraction to orient polarized cell behaviors during *Drosophila* wing morphogenesis

**DOI:** 10.1016/j.cub.2024.11.058

**Published:** 2024-12-20

**Authors:** Larra Trinidad, Alexander G. Fletcher, David Strutt

**Affiliations:** 1 School of Biosciences, University of Sheffield, Firth Court, Sheffield, S10 2TN, UK; 2 School of School of Mathematical and Physical Sciences, University of Sheffield, Hicks Building, Sheffield S3 7RH, UK

## Abstract

During tissue morphogenesis, an interplay of biochemical pathways and mechanical cues regulates polarized cell behaviors, the balance of which leads to tissues reaching their correct shape and size.^1–4^ A well-studied example of a biochemical regulator is the highly conserved Fat-Dachsous (Ft-Ds) pathway that coordinates planar polarized cell behaviors and growth in epithelial tissues.^5,6^ For instance, in the *Drosophila* larval wing disc, the Ft-Ds pathway acts via the atypical myosin Dachs to control tissue shape by promoting the orientation of cell divisions primarily in a proximodistal (PD) direction.^7,8^ Here, we investigate interactions between Ft-Ds planar polarity and mechanical forces in the developing *Drosophila* pupal wing. We show that in the early stages of pupal wing development (16−18 h after puparium formation), anteroposterior (AP)-oriented cell divisions and T1 transitions are controlled by the Ft-Ds pathway acting via Dachs. Shortly there-after, PD-oriented tissue tension is induced across the wing blade by the process of hinge contraction. This opposes the control of Dachs over polarized cell behaviors in a tug-of-war fashion, resulting in more PD-oriented cell divisions and T1s. Furthermore, increased PD tissue tension stabilizes Ft along PD-oriented junctions, suggesting that biomechanical feedback on the Ft-Ds pathway resists the effects of hinge contraction on cell shape. We also show that loss of Dachs results in increased myosin-II stability at cell junctions, revealing compensatory interactions between these two myosins. Overall, we propose that Ft-Ds pathway function constitutes a mechanism whereby tissues are buffered against mechanical perturbations.

## Graphical abstract

**Graphical abstract:**
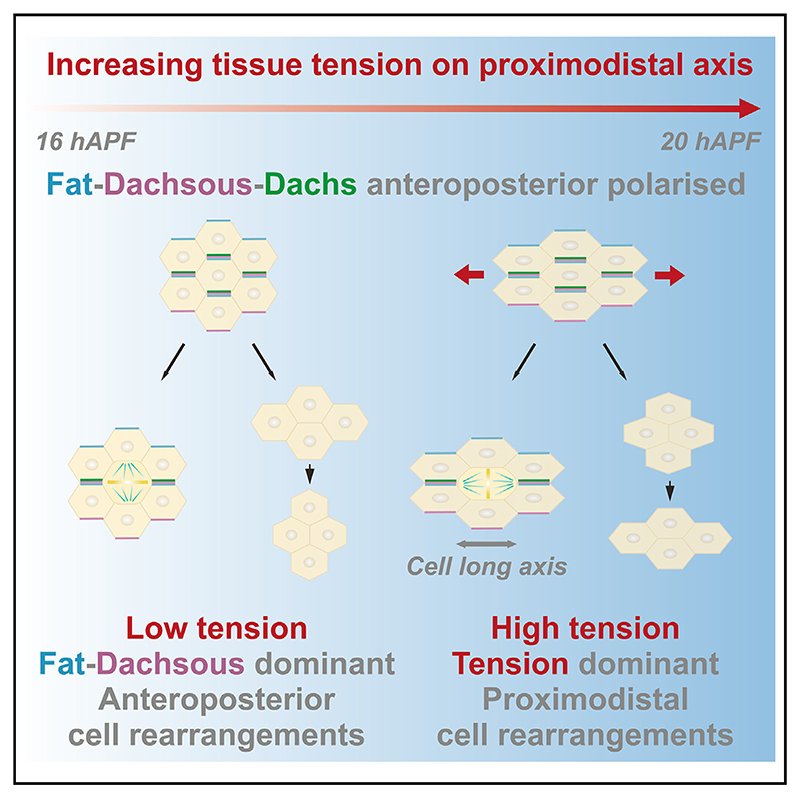

## Results

### The Ft-Ds pathway promotes AP-oriented cell divisions and T1 transitions in pupal wings prior to hinge contraction

**Figure 1:**
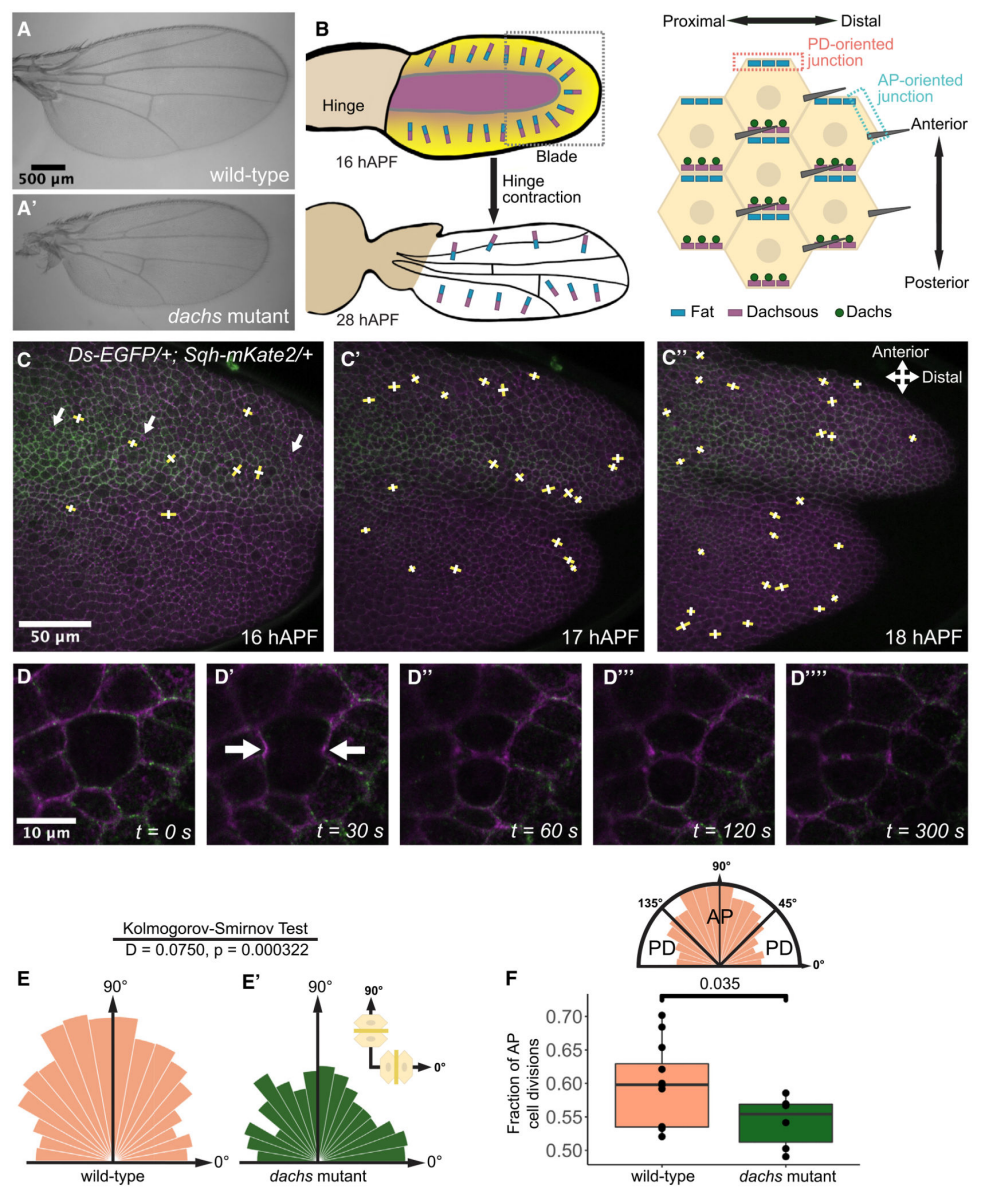
The Ft-Ds pathway controls AP-oriented cell divisions in 16−18 hAPF wild-type wings (A and A′) Wild-type (*w^1118^*) (A) and *dachs* mutant (*d^GC13^*/*Df(2L)BSC201*) (A′) adult wings. (B) Pupal wing cartoons. Top-left: 16 hAPF, Four-jointed gradient (yellow) and Ds expression (purple), and Ft-Ds planar polarity vectors in blue-purple. Gray box indicates region of interest (ROI) for imaging. Bottom-left: hinge contraction elongates wing blade until 28 hAPF. Right: in wing posterior, at the cellular level, Ft is localized along anterior cell junctions and Ds and Dachs along posterior junctions. (C−C″) Video stills of wild-type wing from 16 to 18 hAPF. Ds-EGFP (green) and Sqh-mKate2 (magenta). Yellow lines show new cell junctions formed after cell division, and white lines show defined cell division angle. White arrows indicate sensory organ precursor (SOP) cells (C) used to orient the wing axis (0°). All images distal to anterior cross-vein. (D−D””) A dividing cell rounds up (D), Sqh-mKate2 (magenta) moves to cleavage furrow (D′, arrows), and two daughter cells form (D″−D””). (E and E′) Circular histograms showing distribution of cell division orientation in wild-type from 16 to 18 hAPF (*N* = 12 pupae, *n* = 2,446 divisions) and *dachs* mutants (*N* = 6 pupae, *n* = 1,133 divisions) with 10° interval bins. Two-sample Kolmogorov-Smirnov test performed to compare distributions. (F) Quartile boxplots showing AP-to-PD ratios (angles between 45° and 135° defined as AP-oriented) of cell divisions in wild-type and *dachs* mutants. *p* value calculated using Student’s t test. (See also Figure S1.

Ft and Ds are atypical cadherins that bind in *trans* at cell junctions and provide cellular polarity cues that control tissue shape.^5,6^ Loss of function for either results in shorter, rounder wings^9,10^ (Figure S1A), while loss of their effector Dachs—an atypical myosin—results in smaller, narrower wings^11^ (Figure 1A). Dachs promotes increased junctional tension^8,12^ and, during wing disc development, acts downstream of Fat-Dachsous (Ft-Ds) to control cell division orientation,^8^ while in the pupal notum, Ft-Ds-Dachs regulate polarized cell rearrangements.^12^

Tissue morphogenesis involves an interplay between biochemical and mechanical cues.^1–4^ To learn more about how Ft-Ds-Dachs regulation of cell behaviors is influenced by tissue mechanics, here, we study their function in the developing pupal wing epithelium. During pupal wing morphogenesis, the hinge begins contracting from 16 h after puparium formation (hAPF)^13,14^ (Figure 1B), placing wing-blade cells under anisotropic tension along the proximodistal (PD) axis, resulting in oriented cell elongation, division, and rearrangement in the PD axis.^13,14^ In parallel, Ft-Ds-Dachs localize asymmetrically with anteroposterior (AP)-oriented planar polarity within cells (Figure 1B),^15^ consistent with promoting AP-oriented cell behaviors.

We imaged the distal pupal wing (distal to anterior cross-vein) (Figure 1B, gray box) between 16 and 18 hAPF, at the onset of hinge contraction, when cell divisions are frequent.^13,14^ We used Ds-EGFP to follow Ft-Ds-Dachs polarity (because Dachs follows Ds polarity^12,16^) (Figures 1C and 1D) and Sqh-3xmKate2 to reveal myosin-II distribution^17^ (Sqh is *Drosophila* myosin-II regulatory light chain). Myosin-II accumulation on cell junctions in the developing wing correlates with junctional tension^18–20^; hence, its polarity orientation can reveal tissue tension orientation. As previously reported,^15^ at 16 hAPF, Ds (hence Ft and Dachs) was enriched on PD-oriented junctions, resulting in AP-oriented polarity (Figure S1B). Sqh polarity also appeared weakly AP oriented (Figure S1C), although laser ablation assays suggest that tissue tension is minimal at this stage (see Figure 3C), consistent with previous reports.^13,21,22^

In wild-type wings at 16−18 hAPF, we found that cell divisions were predominantly AP oriented (Figures 1E and 1F). However, in *dachs* mutant wings, the fraction of AP-oriented cell divisions was significantly reduced (Figures 1E′ and 1F). *ft* and *ds* null mutant pupal wings were not flat enough to image due to Dachs misregulation and tissue overgrowth,^11,12,16^ but in hypomorphic mutants, we also observed weak cell division orientation defects (Figures S1D−S1H).

**Figure 2:**
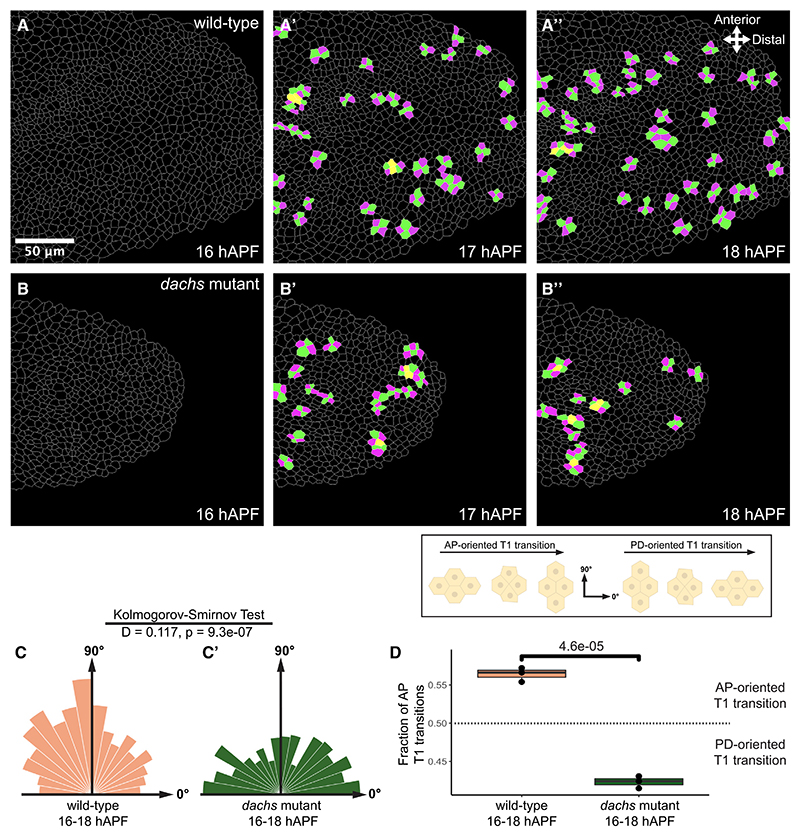
The Ft-Ds pathway controls AP-oriented T1 transitions in 16−18 hAPF wild-type wings (A−B″) Video stills of wild-type (*Ds-EGFP/+; Sqh-mKate2/+*) (A−A″) and *dachs* mutant wings (*Ds-EGFP, Df(2L)BSC201/d^GC13^*; *Sqh-mKate2/+*) (B−B″) were segmented, and T1 transitions were tracked. Magenta cells have lost old contacts, and green cells formed a new junction between them. Yellow cells have lost and gained contacts (neighboring T1s). (C and C′) Circular histograms showing distribution of angles of new cell junctions formed after T1 transitions from 16 to 18 hAPF in wild-type (C) (*N* = 3 pupae, *n* = 1,419 T1s) and *dachs* mutants (C′) (*N* = 3 pupae, *n* = 864 T1s). (D) Quartile boxplots showing AP-to-PD ratios of T1 transitions in wild-type and *dachs* mutants. *p* value was calculated using Student’s t test.

In wild-type wings at 16−18 hAPF, we also saw an enrichment of AP-oriented T1 transitions (neighbor exchanges between four cells, in which new AP-oriented junctions form) (Figures 2A, 2C, and 2D), as previously reported.^13,14^ Notably, in *dachs* mutant wings from 16 to 18 hAPF, T1 transitions were significantly more PD oriented (Figures 2B−2D).

Overall, our results support the conclusion that planar polarized Dachs localization promotes AP-oriented cell divisions and cell rearrangements in the pupal wing prior to hinge contraction (Figure 4Hi), consistent with its functions in the wing disc and pupal notum.^8,12^

### PD-oriented tension and AP-oriented Ft-Ds planar polarity compete to control oriented cell behaviors

**Figure 3:**
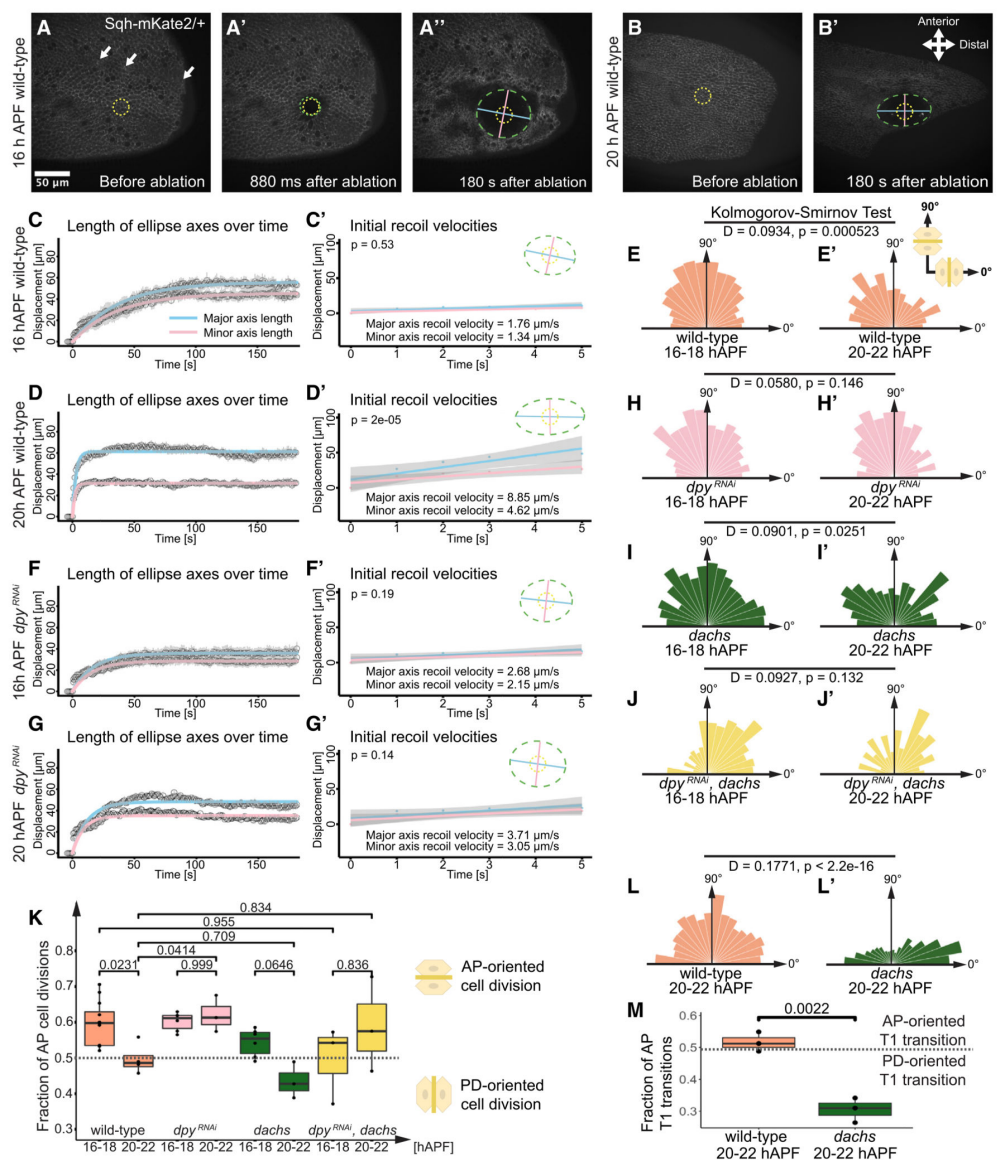
Increased PD tension from hinge contraction controls PD-oriented cell divisions in 20−22 hAPF wings (A−B′) Stills from time-lapses where circular ablation^14^ was performed below second SOP cell in 16 hAPF (A−A″) and 20 hAPF (B and B′) wild-type wings showing Sqh-mKate2, pre-ablation (A and B), 880 ms post-ablation (A′), and 180 s post-ablation (A″ and B′). Arrows indicate SOP cells. Yellow circle (A and B) is ablation position. Tracked ellipse is green, major axis length is blue, and minor axis length is pink. (C and D) Ellipse axes length (major axis blue and minor axis pink). Curves are nonlinear least squares (NLS) fitting of length of ellipse axes over 180 s post-ablation in 16 hAPF (C) and 20 hAPF (D) wild-type wings. Error bars are standard errors of mean (SEM) (*N* = 7 pupae 16 hAPF, *N* = 11 pupae 20 hAPF). (C′ and D′) Initial recoil velocities for major and minor axes (first 5 s) in 16 hAPF (C′) and 20 hAPF (D′) wild-type wings. Shaded bands display the confidence intervals (0.95). Student’s t test performed to compare average major and minor recoil velocities (*N* = 7 pupae 16 hAPF, *N* = 11 pupae 20 hAPF). (E and E′) Circular histograms showing distribution of cell division orientation in wild-type wings 16−18 hAPF (E) (*N* = 12 pupae, *n* = 2,446 divisions) and 20−22 hAPF (E′) (*N* = 4 pupae, *n* = 586 divisions). (F−G′) Curves showing the length of major and minor axes in 16 hAPF (F) and 20 hAPF (G) *dpy^RNAi^* (*nub-GAL4, dpy^RNAi^/+; Sqh-mKate2/+*) wings. Initial recoil velocities for major and minor axes in 16 hAPF (F′) and 20 hAPF (G′) *dpy^RNAi^* wings, *p* values calculated using Student’s t test. (H−J′) Circular histograms showing distribution of cell division orientation in *dpy^RNAi^* wings 16−18 hAPF (H) (*N* = 6 pupae, *n* = 880 divisions) and 20−22 hAPF (H′) (*N* = 3 pupae, *n* = 696 divisions), *dachs* mutants 16−18 hAPF (I) (*N* = 6 pupae, *n* = 1,133 divisions) and 20−22 hAPF (I′) (*N* = 3 pupae, *n* = 354 divisions), and *dpy^RNAi^*, *dachs* wings 16−18 hAPF (J) (*N* = 3 pupae, *n* = 336 divisions) and 20−22 hAPF (J′) (*N* = 3 pupae, *n* = 298 divisions). (K) Quartile boxplots comparing AP-to-PD ratio of cell divisions using ANOVA with Tukey-Kramer multiple comparisons test (see Table S1). Columns 2 (20−22 hAPF wild type), 5 (16−18 hAPF *dachs*), 7 (16−18 hAPF *dpy^RNAi^*, *dachs*), and 8 (20−22 hAPF *dpy^RNAi^*, *dachs*) show intermediate values with negligible AP/PD bias. (L and L′) Circular histograms showing distribution of angle of new cell junctions formed after T1 transitions 20−22 hAPF in wild-type (L) (*N* = 3 pupae, *n* = 2,427 T1s) and *dachs* mutants (L′) (*N* = 3 pupae, *n* = 1,233 T1s). (M) Quartile boxplots showing AP-to-PD ratios of T1 transitions in wild-type and *dachs* mutants. *p* value calculated using Student’s t test. See also Figures S2 and S3.

We next investigated the effect on polarized cell behaviors of the PD-oriented tension produced by hinge contraction. We first confirmed the expected increase in tissue tension^13,14,19,21^ by performing circular ablation assays on wild-type wings (Figures 3A and 3B). At the onset of hinge contraction at 16 hAPF, tissue tension appeared isotropic (Figure 3C) but became highly PD oriented at 20 hAPF (Figure 3D). Furthermore, at 20 hAPF we found that cell division orientation was more PD-oriented as compared with 16−18 hAPF (Figures 3E and 3K; see also Aigouy et al.^13^ and Etournay et al.^14^).

We then asked whether releasing PD-oriented tissue tension might restore AP-oriented cell behaviors. The pupal wing is tethered to the overlying cuticle by the extracellular matrix protein Dumpy (Dpy), and Dpy loss reduces PD tissue tension produced by hinge contraction.^14,23^ We expressed *dpy-RNAi* ubiquitously, resulting in reduced PD-oriented tissue tension at 20 hAPF (Figures 3F and 3G; see also Figures S2A and S4C) and cell divisions being more AP-oriented (Figures 3H and 3K). Importantly, Ft-Ds remained AP oriented in *dpy-RNAi* wings (Figures S2B and S2C).

Based on these results, we speculated that AP-oriented Ft-Ds-Dachs and PD-oriented tissue tension might compete to regulate cell division orientation. To test this, we first analyzed 20−22 hAPF *dachs* mutant wings and found that cell divisions were now more PD-oriented (Figures 3I and 3K), and tissue tension remained PD oriented (Figures S2D and S2E). We then looked at cell division orientation in *dachs* mutant wings also expressing *dpy-RNAi* to reduce PD-oriented tissue tension and found that cell divisions were now unpolarized at both 16−18 and 20−22 hAPF (Figures 3J and 3K).

We also investigated the control of T1 transitions by Ft-Ds-Dachs from 20 to 22 hAPF. Consistent with the effects on cell division orientation, in *dachs* mutant wings, T1 transitions became more PD oriented (Figures 3L and 3M). Moreover, while loss of Dachs did not alter overall cell elongation (Figure S3A), it did cause the axis of elongation to become more tightly PD oriented (Figures S3B and S3C). Thus, these results are consistent with Dachs activity normally opposing the effects of hinge contraction on oriented cell shape and behaviors.

**Figure 4:**
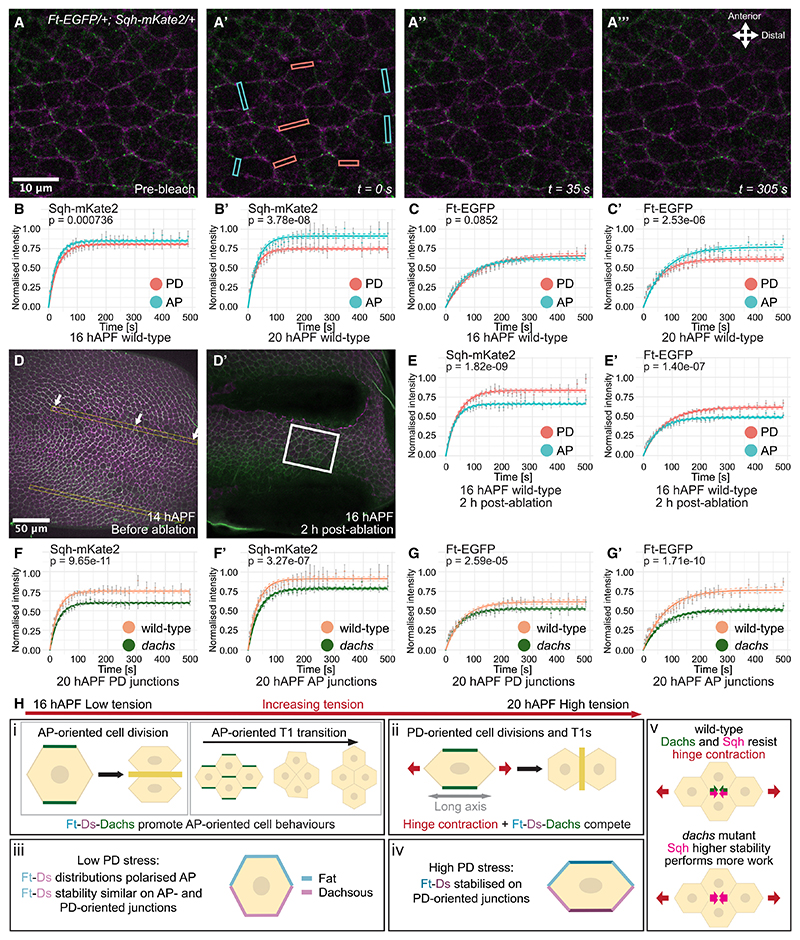
Increased junctional tension stabilizes Sqh and Ft (A−A%) FRAP on Sqh-mKate2 (magenta) and Ft-EGFP (green) performed below second SOP in 16 hAPF wild-type wings. Images pre-bleach (A), immediately post-bleaching (pink and blue boxes indicate bleached ROIs) (A′), and 35 s (A″) and 305 s (A%) post-bleaching. For all FRAP experiments, 4 vertical junction and 4 horizontal junction ROIs chosen per pupa, and experiment repeated over multiple pupae to provide biological replicates. (B−C′) One-phase exponential NLS fitting of average normalized intensity for post-bleaching recovery in wild-type pupal wings for Sqh-mKate2 (B) and Ft-EGFP (C) 16 hAPF (*N* = 11), and for Sqh-mKate2 (B′) and Ft-EGFP (C′) 20 hAPF (*N* = 9), where error bars represent SEM, and dashed lines show confidence intervals. Blue and pink curves show recovery along AP-oriented and PD-oriented junctions, respectively. (D and D′) 14 hAPF wild-type pupal wing pre-ablation (D) and 2 h post-ablation (D′). Yellow boxes (D) show where two parallel cuts were made, and white box (D′) indicates ROI, where FRAP was performed. (E and E′) One-phase exponential NLS fitting of average normalized intensity for post-bleaching recovery for Sqh-mKate2 (E) and Ft-EGFP (E′) in wings where laser cuts performed (*N* = 8). (F−G′) One-phase exponential NLS fitting of average normalized intensity for post-bleaching recovery for Sqh-mKate2 (F and F′) and Ft-EGFP (G and G′) in wildtype (*N* = 9) and *dachs* mutant (*N* = 11) wings at 20 hAPF along PD-oriented junctions (F and G) and AP junctions (F′ and G′). Orange and green curves denote recovery on wild-type and *dachs* mutant junctions, respectively. *p* values comparing fluorescence recovery plateaus (*y*_*max*_ values) in FRAP experiments calculated using unpaired t tests (Table S2). (H) Model: (i) At 16 hAPF, Dachs (green) promotes AP-oriented cell divisions and T1 transitions. (ii) When hinge contraction induces sufficient PD stress, cell divisions and T1 transitions become more PD-polarized. (iii) At low PD stress, Ft-Ds (blue, purple) polarity is AP oriented, but their stability is similar on all junctions. (iv) Under sufficient PD stress, Ft-Ds are stabilized along PD-oriented junctions. (v) Dachs, along with Sqh (magenta), resists hinge contraction. Sqh compensates for Dachs loss by becoming more stable. See also Figure S4 and Table S2.

Our data support the hypothesis that AP-oriented Ft-Ds planar polarity and PD-oriented tissue tension compete in a tug-of-war to control cell behaviors. At 16−18 hAPF Ft-Ds dominate to promote AP-oriented cell behaviors, but at 20−22 hAPF, PD-oriented tension competes to re-orient cell behaviors along the PD axis (Figure 4Hii).

Finally, we looked at the effects of *dachs* loss on overall wing shape. While wild-type pupal wings get fatter along the AP axis between 16 and 24 hAPF, *dachs* mutant wings became narrower (Figures S3D and S3E), consistent with reduced AP-oriented cell rearrangements.

### Junctional tension stabilizes Ft

Because Ft-Ds are preferentially localized to PD-oriented cell junctions, which experience higher junctional tension due to hinge contraction,^13,14,19,21^ we wondered whether junctional tension might reduce turnover and increase stability of Ft-Ds complexes. Such stabilization of Ft-Ds AP-polarity by PD-oriented tension could act to oppose effects of PD-tension on oriented cell behaviors.

We used fluorescence recovery after photobleaching (FRAP) on Sqh-mKate2 as a sensitive assay for inferring junctional tension because increased junctional tension reduces Sqh turnover.^20,24^ At 16 hAPF, fluorescence recovery of Sqh-mKate was similar on PD-oriented and AP-oriented junctions (Figures 4A and 4B), consistent with our circular ablation data failing to detect anisotropy in tissue stress at this stage (Figure 3C). FRAP assays at 16 hAPF further revealed that Ft had similar stability on PD-oriented and AP-oriented junctions (Figure 4C). At 20 hAPF, Sqh was noticeably more stable on PD-oriented junctions (Figure 4B′), implying higher junctional tension, in agreement with our circular ablation results (Figure 3D) and previous findings.^13,14,19,21^ Notably, Ft also became more stable along PD-oriented junctions at 20 hAPF (Figure 4C′).

To confirm that increased junctional tension stabilizes Ft, we reoriented tissue tension along the AP axis by performing two parallel PD-oriented laser cuts and waiting so that wound-healing contractile rings pull the tissue along the AP axis^25,26^ (Figure 4D). Circular ablation confirmed an increase in AP-oriented tension (Figures S4A and S4B), and both Ft and Sqh became more stable along AP-oriented junctions (Figures 4E and 4E′).

Finally, we reduced PD-oriented junctional tension using *dpy-RNAi*. Sqh retained weak AP polarity (Figure S2A) with slightly higher stability on PD-oriented junctions (Figure S4C, compare Figure 4B′). Interestingly, Ft now appeared marginally more stable on AP-oriented junctions (Figure S4C′). Taken together, these results show that Ft junctional stability to Sqh) is mechanoresponsive to the PD tension provided by hinge contraction (Figures 4Hiii and 4Hiv).

### Loss of Dachs stabilizes Sqh and Ft

Because Dachs promotes junctional tension,^8,12^ we wondered whether stability of the mechanosensitive actomyosin network would be altered upon Dachs loss. Interestingly, we found that Sqh became more stable in 20 hAPF *dachs* mutants (Figures 4F and 4F′), implying higher junctional higher tension being generated by Sqh. This suggests that Sqh is recruited to compensate for the lack of Dachs, and that Dachs (alongside Sqh) normally counteracts the tissue tension from hinge contraction (Figure 4Hv).

We previously found that Ft-Ds polarity magnitude increases upon Dachs loss.^16^ We wondered whether this effect on Ft-Ds polarity might occur via effects on Ft or Sqh stability. Notably, we found that Ft was more stable at junctions in *dachs* mutant wings at 20 hAPF (Figure 4G) where Sqh is stabilized. However, we also saw stabilization of Ft, but only negligible differences in Sqh in *dachs* mutant wings at 16 hAPF (Figures S2D and S4D). Our data show that Dachs is not only a downstream effector of Ft-Ds but also modulates Ft-Ds stability, possibly via direct effects on actomyosin networks.^27^

## Discussion

Here, we report that during *Drosophila* pupal wing development, a tug-of-war exists between Ft-Ds planar pathway and tissue stress in controlling oriented cell behaviors. At earlier stages, the Ft-Ds pathway (via Dachs) promotes AP-oriented cell divisions and T1 transitions; however, when sufficient PD tissue stress has been generated by hinge contraction, cell behaviors become more PD-oriented (Figures 4Hi and 4Hii). Notably, something similar occurs in the wing disc, where Dachs promotes PD-oriented cell divisions in the distal wing pouch,^8^ but increased tension at the periphery elongates cells circumferentially, causing reorientation of cell divisions from PD to radial.^18,28^ We further show that tissue mechanics feed back onto Ft-Ds, whereby junctional tension promotes Ft junctional stability (Figure 4Hiv). We propose that tension-dependent effects on Ft stability constitute a feedback mechanism whereby the Ft-Ds pathway buffers against effects of tissue tension on oriented cell behaviors.

How Dachs promotes AP-oriented cell behaviors remains unclear. Because Dachs promotes junctional tension, one possibility is that Dachs enrichment on PD-oriented junctions results in junction contraction and AP-oriented cell elongation, in turn, promoting AP-oriented cell behaviors, e.g., following Hertwig’s rule.^29^ However, cells are largely PD elongated prior to hinge contraction at 16 hAPF (Figures S3A and S3B), possibly due to microtubule-generated forces,^22^ yet cell rearrangements are largely AP-oriented. A similar paradox exists in the distal wing pouch,^8^ where it has been suggested that Dachs promote a change in the axis of cell elongation just at the point of cell division.

It is also not known how Ft-Ds might sense junctional tension. Tension could directly induce conformational changes in Ft/Ds, altering Ft-Ds *trans*-dimers stability. Alternatively, mechanosensing could depend on the actomyosin network and be mediated through the actin-binding ability of Dachs.^27^

In summary, we find that Ft-Ds planar polarity competes with mechanical tension to control oriented cell behaviors. Furthermore, Ft-Ds are stabilized by tension, constituting a possible mechanism whereby tissues are buffered against mechanical perturbations.

## Resource Availability

### Lead contact

Further information and requests for resources and reagents should be directed to and will be fulfilled by the lead contact, David Strutt (d.strutt@sheffield.ac.uk).

### Materials availability

Fly strains used in this paper will be shared by the lead contact upon request.

## Star⋆ Methods

Detailed methods are provided in the online version of this paper and include the following:

- KEY RESOURCES TABLE
- EXPERIMENTAL MODEL
- METHOD DETAILS
  ○ Pupal wing dissection and mounting
  ○ Image acquisition
  ○ FRAP experiments
  ○ Laser ablation
  ○ Adult wing dissection and mounting
- QUANTIFICATION AND STATISTICAL ANALYSIS
  ○ Image segmentation and polarity measurements
  ○ Measuring cell divisions
  ○ Measuring T1 transitions
  ○ Circular ablation analysis
  ○ Statistics
  ○ FRAP experiments

## Star⋆ Methods

### Key Resources Table

**Table:** 

REAGENT or RESOURCE	SOURCE	IDENTIFIER
Experimental models: *Drosophila melanogaster* strains		
*w^1118^*	Bloomington Drosophila Stock Center	FlyBase: FBgn0003996
*ft-EGFP*	Hale et al.^30^	FlyBase: FBal0385338
*ds-EGFP*	Brittle et al.^16^	FlyBase: FBal0344517
*ft^1^*	Bloomington Drosophila Stock Center	FlyBase: FBst0000304
*ft^G-rv^*	Bloomington Drosophila Stock Center	FlyBase: FBst0001894
*ds^1^*	Clark et al.^31^	FlyBase: FBal0003119
*ds^UA071^*	Adler et al.^32^	FlyBase: FBal0089339
*d^GC13^*	Mao et al.^11^	FlyBase: FBal0128007
*Df(2L)BSC201*	Bloomington Drosophila Stock Center	FlyBase: FBab0044944
*sqh∷sqh-3xmKate2*	Pinheiro et al.^17^	FlyBase: FBal0358816
*nub-GAL4*	Bloomington Drosophila Stock Center	FlyBase: FBtp0009119
*dpy^RNAi^* (GD4443)	Vienna *Drosophila* RNAi Center	FlyBase: FBal0209383
Software		
NIS Elements AR version 4.60	Nikon	N/A
FIJI version 2.9.0	https://fiji.sc ^33^	PMCID: PMC3855844
Tissue Analyzer version	https://grr.gred-clermont.fr/labmirouse/software/WedPA/ ^34^	PMID: 27730585
RStudio version 3.6.1	http://www.rstudio.com/	N/A
QuantifyPolarity version 2.0	Tan et al.^35^	PMCID: PMC8451067
Reagents		
Heptane glue	Heptane glue was made by placing Sellotape in heptane.	N/A
Gary’s Magic Mountant (GMM)	50% Canada balsam, 50% methylsalicylate	N/A

### Experimental Model

*Drosophila melanogaster* lines were grown on standard cornmeal/agar/molasses media at 25°C. There are no known differences in the physical and molecular mechanisms of planar polarity in male and female flies, thus flies were not distinguished based on sex for pupal wing experiments. For adult wings, examples from males are shown. Fly strains are described in FlyBase as indicated in the key resources table.

## Method Details

### Pupal wing dissection and mounting

Pupal wing dissection for imaging was performed as previously reported.^36^ Briefly, pupae were placed on double-sided tape (Sellotape), dorsal side up. Using a razor blade and some forceps, a small piece of cuticle was removed from above the pupal wing. The pupae were then floated off the double-sided tape with distilled water and allowed to dry. The pupa was mounted on a glass-bottomed dish coated with heptane glue so that the exposed wing faced the glass.

### Image acquisition

For live imaging experiments, an inverted Nikon A1R GaAsP confocal microscope with a Nikon 60x oil apochromatic objective lens oil (NA=1.4) was used. The pinhole was set to 1.2 Airy Units (AU). Images were taken in the distal region of the wing with 1024 × 1024 pixels per z-slice and 210 nm pixel size. A 488 nm laser and a 561 nm laser were used at 0.6% laser power and 100 gain settings. For the long timelapse imaging experiments, images were taken at 10-minute intervals for two hours. For each image, z-stacks were taken with ∼40 slices per stack at 150 nm intervals.

### FRAP experiments

FRAP assays were performed as described previously.^36^ All FRAP experiments were performed in two colours at 5x zoom producing a region of 512 × 512 pixels with a pixel size of 80 nm. For pre- and post-bleach images, a 488 nm laser and a 561 nm laser were used at 0.6% laser power and 100 gain settings. Eight 1 μm x 0.5 μm regions of interest (ROIs) (always four horizontal and four vertical junctions) were selected per wing and bleached at one laser pass using the 488 nm and 568 nm lasers at 80% power. Three pre-bleach images were captured with no delay as well as an immediate post-bleach image. Five fluorescence recovery images were captured every 5 s, then ten images were captured every 10 s, ten images every 15 s and seven images every 30 s. An image with both laser powers set at 0 was also taken after each experiment to account for the background fluorescent noise.

### Laser ablation

Laser ablation experiments were performed using a Nikon W1 Spinning Disk Confocal equipped with a pulsed ultraviolet (UV) laser, at a frequency of 500 Hz, a dwell time of 20 μs^37^ and 10% laser power. For the circular ablations, the ROI of diameter of 24.3 mm is under the second SOP cell. For the two-parallel horizontal ablations, we performed laser ablations along the line of the SOP cells and a parallel line 100 μm below. For the circular ablation between the two-parallel cuts, a circular ROI of diameter 10 μm was used. Live-imaging was performed with a recording rate of 1 frame per second using a 561 nm laser at 50% laser power. For the circular ablation with diameter of 24.3 μm, images were taken for 5 s before ablation, and after the t=0 frame, the tissue was ablated for 120 ms, then imaged after a further 880 ms t=1 to t=180. The t=1 to t=6 frames were analysed to measure the initial recoil velocity. For the circular ablation with diameter of 10 μm, the tissue was ablated for 65.7 ms, then imaged after a further 934.3 ms t=1 to t=180, and analysed as above.

### Adult wing dissection and mounting

Male adult wings were dehydrated in isopropanol, mounted in Gary’s Magic Mountant (GMM, 50% Canada Balsam, 50% methyl-salicylate), and incubated overnight on a 60°C hot plate. Wings were imaged at 5x magnification.

## Quantification and Statistical Analysis

### Image segmentation and polarity measurements

In all image analyses, FIJI was used to measure the angle of the line of sensory organ precursor (SOP) cells. All angle measurements were normalised against this angle such that the line formed from the SOP cells was taken as 0°. Average projections were performed using FIJI, and cell boundaries were segmented using Tissue Analyzer.^34^ QuantifyPolarity^35^ was then used for polarity and cell shape measurements. For polarity measurements, the Principal Component Analysis (PCA) method was used since this method is less sensitive to cell eccentricity than the Ratio or the Fourier Series methods.^35^ In brief, the PCA method compresses cells into regular shapes and normalises the intensities. This method computes the angle that produces the largest variance of weighted intensities from the centroid of the cell and defines this as the polarity angle of that cell. The extent of the variance is then defined as the polarity magnitude. Coarse-grained polarity vectors can also be plotted, and for this work, single cell polarity vectors were averaged over squares of 3 × 3 cells, which reflects the polarity strength and orientation within a group of cells. For the polarity histograms, cell-by-cell polarity angles from QuantifyPolarity were grouped into bins of size 10° and weighted by the respective cell-by-cell polarity magnitude (i.e., the height of each cell-by-cell polarity angle in each bin is incremented by the cell-by-cell polarity magnitude). Data from multiple wings were then combined and a circular histogram was plotted using R. This way, the length of each bin represents the average magnitude of polarity within the bin.

### Measuring cell divisions

For cell division orientation analyses, the angle of the new boundary formed after cell division was manually identified by eye and a line was drawn on the new cell junction. The angle was measured using FIJI. 90° was added to this angle, which is defined as the angle of cell division. The angles were grouped into bins of size 10° and a circular histogram was plotted using R.

### Measuring T1 transitions

Tissue Analyzer^34^ was used to track cell movements throughout the tissue; however, the cell tracks required manual correction to ensure precise tracking. Corrected cell tracking maps were then used to measure the T1 transition orientation in wild-type wings from 16-18 and 20-22 hAPF. Tissue Analyzer recognises four-way vertices and colour-codes the cells that have undergone a junctional rearrangement (4-way vertex vs bond cutoff of 4). Some mistakes in identifying T1 transitions occur during the tracking (especially when there are neighbouring cell divisions). Hence, the individual T1 transitions were manually checked to ensure no tracking errors were made. The angle of the new cell junction was measured using FIJI. Circular histograms were then plotted to show the distribution of the angles of newly formed junctions.

### Circular ablation analysis

FIJI was used to draw along the vertices around the edge of the ablation and the built-in ‘Measure’ function was then used to find the major and minor axes lengths at each timepoint, and a nonlinear least squares (NLS) fitting of the length of the ellipse axes over 180 s post-ablation was performed. Initial recoil velocity is defined as the change in the length of ellipse major (or minor) axis over time during the first 5 s. A Student’s t-test was used to compare the major and minor axis velocities per genotype.

### Statistics

For statistical comparisons between angle distributions, a two-sample Kolmogorov-Smirnov test was performed^38^ using a built-in R function (https://www.rdocumentation.org/packages/dgof/versions/1.4/topics/ks.test). In brief, the null hypothesis is that the two groups are drawn from the same distribution. The cumulative distribution for each group is compared. Two values are calculated: D, the maximum difference between the two cumulative distributions, and the p-value, the significance level. This test does not assume that the data are sampled from Gaussian distributions or any other defined distributions.

Quartile box plots were plotted, and the data were compared using either Student’s t-tests or ANOVA with Tukey-Kramer multiple comparisons test.

### FRAP experiments

For analysis of each FRAP experiment on a single pupa, the four (horizontal or vertical), ROIs were reselected and four non-bleached ROIs (horizontal or vertical) were individually tracked to measure the intensities at each timepoint. The software package R was used to correct the data for acquisition bleaching and normalise against pre-bleach values such that pre-bleach intensities were set to 1 and post-bleach intensities were set to 0. Additional checks were performed to ensure the FRAP settings used were suitable so that there was sufficient initial bleaching and acquisition bleaching is less than 25%. The initial bleaching is calculated by measuring the average intensities of the four (horizontal or vertical) FRAP ROIs before bleaching $\underline{\left(l_{-1}\right)}$ and immediately after bleaching $\underline{\left(l0\right)}$ and using the equation:

$$Initial\;bleaching\;=100-100\left(\frac{I_{0}}{\underline{I_{-1}}}\right).\;$$

In all FRAP experiments performed, initial bleaching was between 50-75%.

To determine the acquisition bleaching, the average intensities of the four unbleached regions at the start $\underline{\left(U_{-1}\right)}$ and at the end of the experiment (*U*_*end*_) were measured. The acquisition bleaching is found by using the following equation:

$$\;Acquisition\;bleaching\;=100(\frac{\underline{U_{-1}}-\underline{U_{end\;}}}{\underline{U_{-1}}-\;background})$$

In all FRAP experiments, the acquisition bleaching was less than 25%.

All intensities at all timepoints for the FRAP ROIs were corrected for acquisition bleaching, here denoted as *A*_*n*_, and background noise by using the following equation:

$$A_{n}=(\frac{\underline{U_{-1}}-\;background\;}{U_{n}-\;background\;})\left(\frac{I_{n}-\;background\;}{I_{0}-\;background\;}\right),$$

where *I*_*n*_ is the intensity of the bleached region at timepoint *n*, so that *I*_0_ is the initial intensity of the region and *U*_*n*_ is the unbleached control intensity at that timepoint.

To normalise the values between 0 and 1, the following equation was used:

$$N(n)=\left(\frac{A_{n}-A_{0}}{1-A_{0}}\right),$$

where *A*_0_ is the intensity (corrected for acquisition bleaching) at time *t = 0* (i.e., immediately after bleaching) and *A*_*n*_ is the intensity (corrected for acquisition bleaching) at time *t= n*.

For each pupa, the average normalised intensities for each timepoint for the four horizontal (or vertical) ROIs were calculated (here n number is the number of ROIs, technical replicates). These were then plotted on a graph of the normalised intensity (*y*) against time (*t*) and a one-phase exponential curve was fitted according to the equation *y* = *y*_*max*_(1 - *e*^1−*α t*^, where *y*_*max*_ is the fluorescence recovery plateau and *α* is the rate constant from which the recovery half-life is calculated (one N). For each genotype, the average normalised intensity for each timepoint was then calculated across multiple pupae and an average one-phase exponential curve was again fitted to determine the final fluorescence recovery plateau (*y*_*max*_) and half-life. Statistical tests were done using the average value per pupa, where N (number of pupae) represents the number of biological replicates.

Confident intervals for the *y*_*max*_ and *α* in the fitted models were calculated using a built-in R function (https://www.rdocumentation.org/packages/stats/versions/3.6.2/topics/confint). *y*_*max*_ values were compared using a Student’s unpaired t-test to calculate the p-values.

A detailed statistical table for all the FRAP fitting data is in Table S2.

Two-phase fits of the data were also performed, to see if more than one mode of recovery (i.e. a fast phase and a slow phase) could be detected. However, statistical differences between the fast phases of recovery between different conditions could not be detected reliably. In some cases, the confidence intervals could not be calculated (‘NA’ values in Table S2), likely due to the data simply not defining the parameter in the two-phase exponential model very well (perhaps due to widely scattered parameter estimates), leading to undefined confidence intervals. These data are included in Table S2.

## Supplementary Material

Supplementary Material

## Data Availability

All data reported in this paper will be shared by they lead contact upon request. R code used for statistical analysis is available at https://github.com/LarraTrinidad/LarraT/tree/codes. Any additional information required to reanalyze the data reported in this paper is available from the lead contact upon request.

## In brief

Morphogenesis involves interactions between biochemical and mechanical cues. Trinidad et al. show that during *Drosophila* pupal wing morphogenesis, Fat-Dachsous planar polarity competes with tissue tension to control oriented cell behaviors. Moreover, junctional tension stabilizes Fat-Dachsous, supporting the existence of biomechanical feedback.

## Highlights

- Fat-Dachsous via Dachs orient anteroposterior cell behaviors in early pupal wings
- Fat-Dachsous competes with hinge contraction in orienting cell behaviors
- Junctional tension stabilizes Fat-Dachsous along proximodistal-oriented junctions
- The myosins Dachs and Sqh exhibit compensatory interactions
