# Supplementary material for: The Fat-Dachsous planar polarity pathway competes with hinge contraction to orient polarized cell behaviors during Drosophila wing morphogenesis
Source: Curr Biol. Author manuscript; Available in PMC 2025 Jan 23. (PMC7617321; doi:10.1016/j.cub.2024.11.058)
Supplement: Supplementary Material [file EMS202683-supplement-Supplementary_Material.pdf]

Current Biology, Volume *35*

Supplemental Information

The Fat-Dachsous planar polarity pathway competes with hinge contraction to orient polarized cell behaviors during *Drosophila* wing morphogenesis

Larra Trinidad, Alexander G. Fletcher, and David Strutt

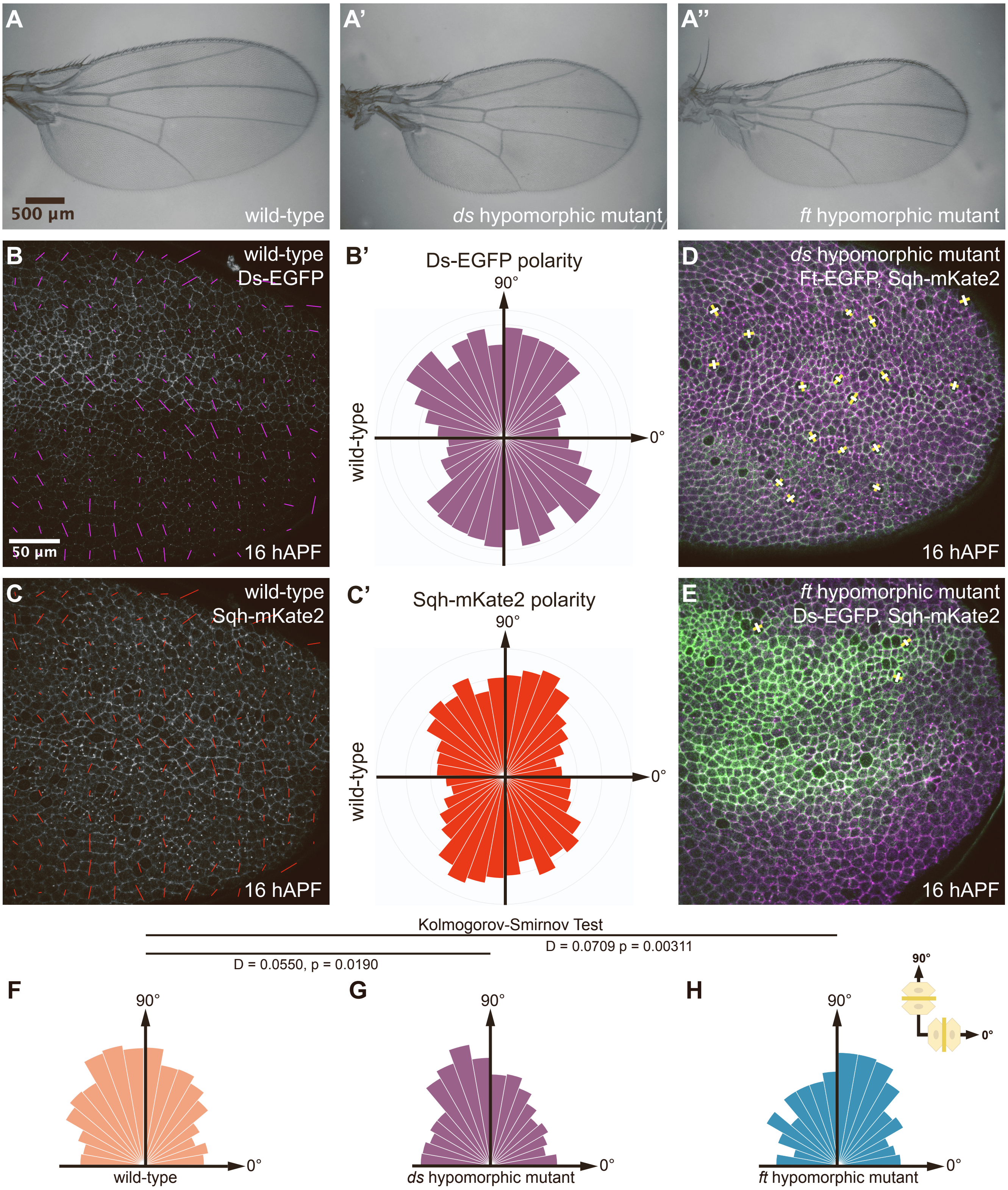

Figure S1. The Ft-Ds pathway controls AP-oriented cell divisions in 16-18 hAPF wild-type wings, related to Figure 1

(A) Adults wings of wild-type (w^{1118}) (A), *ds* hypomorphic mutant (ds^{UA071}/ds^{1}) (A'), and *ft* hypomorphic mutant ($ft^{G\text{-}rv}/ft^{1}$) (A'').

(B-C') Average projection images of Ds-EGFP (B) and Sqh-mKate2 (C) in wild-type wings at 16 hAPF with coarse-grained polarity nematics, where the single-cell polarity vectors were averaged over squared of 3 x 3 cells which

reflects the polarity strength and orientation of a group of cells. Weighted circular diagrams of the cell-by-cell polarities of Ds-EGFP (B') and Sqh-mKate2 (C') in a 16 hAPF wild-type wing (N=3 pupae, n=3473 cells). Sqh shows AP-oriented polarity (increased protein levels on PD-oriented junctions), implying PD-orientation of tissue tension. In addition, we note that Sqh is slightly more stable along the PD-oriented junctions compared to AP-oriented junctions at this stage (see Figure 4B, Table S1) again consistent with PD-oriented tension. However, we are unable to detect significant anisotropy in tissue tension using circular laser ablation at this stage of development (see Figure 3), possibly due to the cellular magnitude of Sqh polarity being relatively weak.

(D,E) Average projection images of Ft-EGFP (D, green) or Ds-EGFP (E, green) and Sqh-mKate2 (magenta) in *ds* (D) and *ft* (E) hypomorphic mutants. Yellow lines show the new cell junctions formed after cell division and white lines show the defined cell division angle.

(F-H) Circular histograms to show the distribution of the orientation of cell division in 16-18 hAPF wild-type wings (N=12 pupae, n=2446 cell divisions), *ds* hypomorphic mutant wings (N=5 pupae, n=921 cell divisions), and *ft* hypomorphic mutant wings (N=4 pupae, n=683 cell divisions). Two-sample Kolmogorov-Smirnov test was performed to compare the distributions.

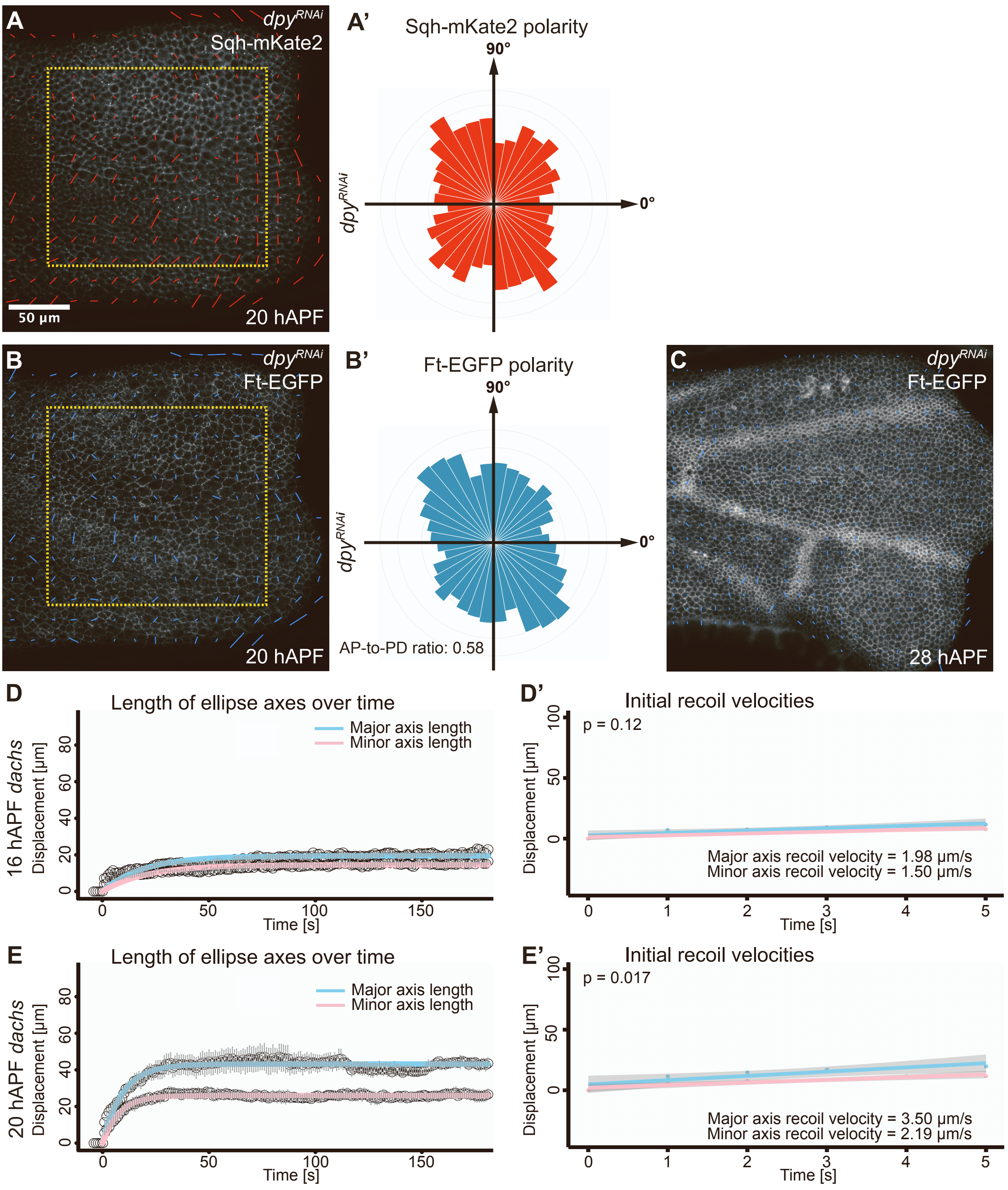

Figure S2. Ft is polarised along the AP axis in *dpy*^{RNAi} wings and *dachs* mutant wings exhibit high PD stress at 20 hAPF, related to Figure 3

(A-C) Average projection images of Sqh-mKate2 (A) and Ft-EGFP (B, C) in *dpy*^{RNAi} (*nub-GAL4, dpy*^{RNAi}/+) wings at 20 hAPF (A, B) or 28 hAPF (C) with coarse-grained polarity nematics, where the single-cell polarity vectors were averaged over squared of 3 x 3 cells which reflects the polarity strength and orientation of a group of cells. Weighted circular histograms for Sqh-mKate2 (A') and Ft-EGFP (B') polarity angles of cells within the yellow box in

20 hAPF *dpy*^{RNAi} wings (N=3 pupae, n=4477 cells). We excluded the polarity measurements of cells near the wing margin to remove boundary effects. At 28 hAPF, Ft-EGFP is still AP-oriented (C).

(D-E'') Circular ablation in 16 hAPF (D-D') and 20 hAPF (E-E') *dachs* mutant wings. The curves are the nonlinear least squares (NLS) fitting of the length of the ellipse axes over 180 s. (D', E') Initial recoil velocities for the major and minor axes. The error bars are the standard errors of the mean. Shaded error bands display the confidence intervals (level 0.95). A Student's t-test was performed to compare the average major recoil velocity and average minor recoil velocity (N=7 pupae for each experiment).

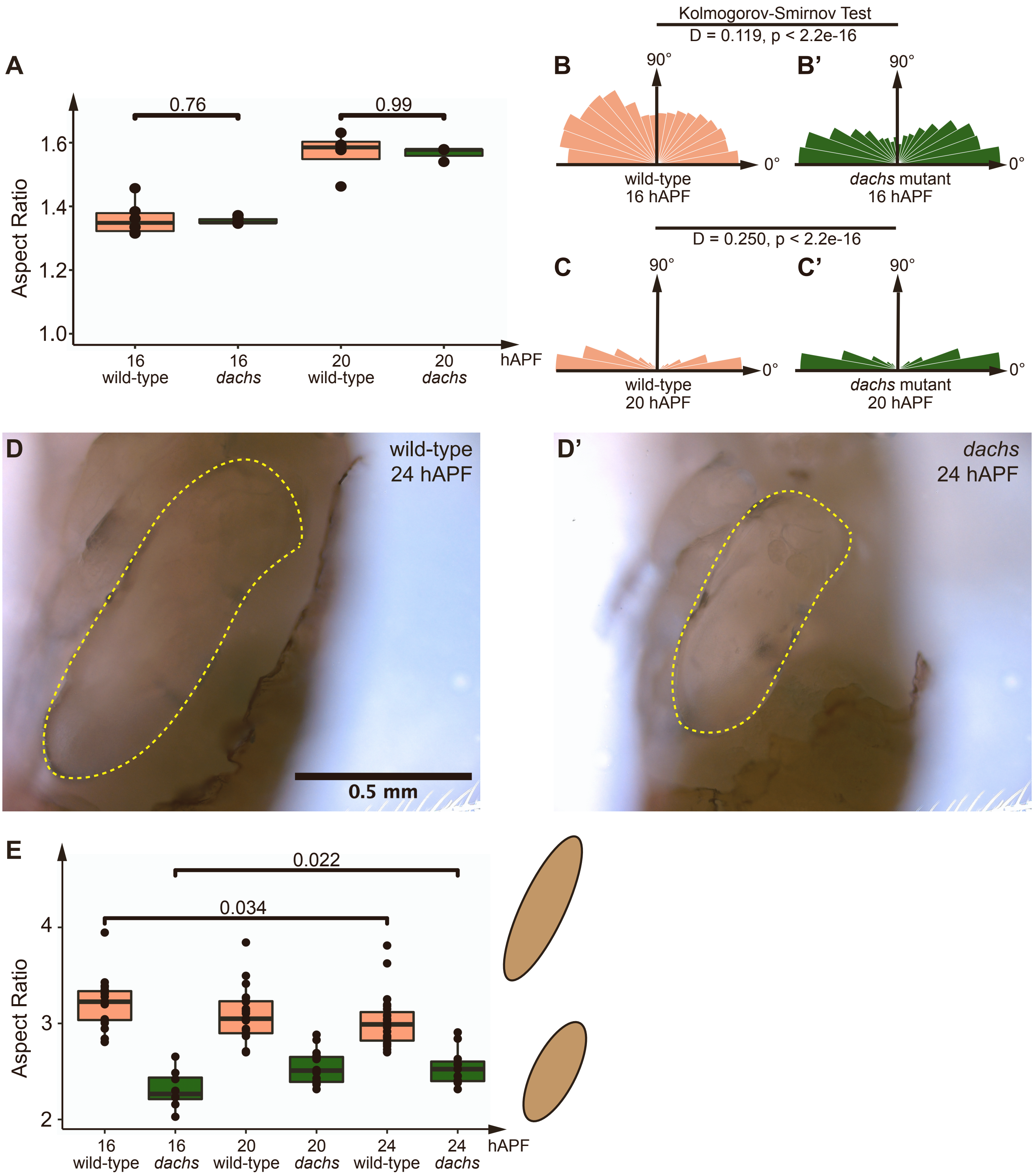

Figure S3. Loss of Dachs affects pupal wing shape and cell orientation, related to Figure 3

(A) Quartile box plots comparing the cell elongation aspect ratio in wild-type and *dachs* mutant wings at 16 hAPF (N=6 pupae for wild-type and N=6 pupae for *dachs*) and 20 hAPF (N=3 pupae for wild-type and N=4 pupae for *dachs*).

(B-C) Rose plots comparing the cell elongation of wild-type and *dachs* mutant wings at 16 hAPF (B-B’) (N=6 pupae, n=6060 cells for wild-type, N=6 pupae, n=5076 cells) and 20 hAPF (C-C’) (N=4 pupae, n=5099 cells for wild-type, N=3 pupae, n=2441 cells).

(D) Images of 24 hAPF wild-type (D) and *dachs* mutant pupae (D’). The pupal wing shape is outlined in yellow.

(E) Quartile box plots show the quantification of pupal wing aspect ratio in wild-type and *dachs* mutants at 16 hAPF (N=19 pupae for wild-type, N=8 pupae for *dachs*), 20 hAPF (N=21 pupae for wild-type, N=18 pupae for *dachs*), and 24 hAPF (N=23 pupae for wild-type, N=11 pupae for *dachs*). P value calculated using a Student’s t-test.

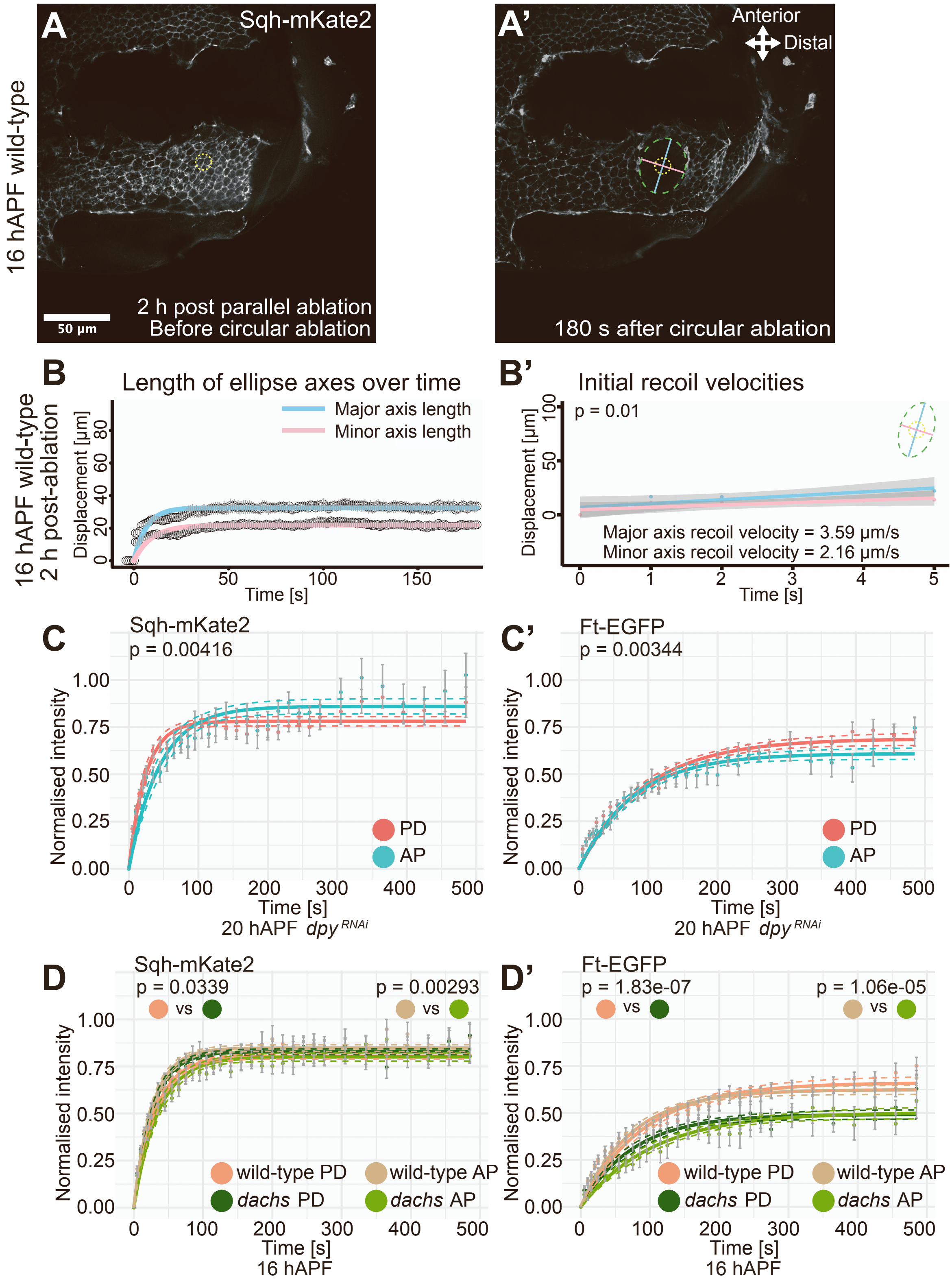

Figure S4. Increased junctional tension stabilises Ft-EGFP and Sqh-mKate2, related to Figure 4

(A-A') Still images from timelapses of 16 hAPF wild-type wings, where circular ablation to assess tissue tension was performed in the region between the two-parallel 14 hAPF horizontal ablations (see Figure 4D-D') using Sqh-mKate2 as a junctional marker, pre-ablation (A) and 180 s post-ablation (A'). Yellow circle is where the UV ablation laser was focused. Tracked ellipse is in green (major axis length is in blue, minor axis length is in pink).

(B) Ellipse axes length (major axis in blue, minor axis in pink). Curves are the nonlinear least squares (NLS) fitting of the length of the ellipse axes over 180 s post-ablation in 16 hAPF wild-type wings (2 h after performing two-parallel horizontal ablations, see Figure 4D-D'). Error bars are standard errors of the mean (SEM) (N=8 pupae).

(B') Initial recoil velocities for major and minor axes (first 5 s) in 16 hAPF wild-type wings (2 h after performing two-parallel horizontal ablations, see Figure 4D-D'). Shaded error bands display the confidence intervals (level 0.95). Student's t-test was performed to compare the average major and minor recoil velocities (N=8 pupae).

(C-C') FRAP on 20 hAPF *dpy*^{RNAi} wings (N=8 pupae). One-phase exponential NLS fittings of the average normalised intensity for the recovery post-bleaching across pupal wings for Sqh-mKate2 (C) and Ft-EGFP (C'), where error bars represent the standard errors of the mean and the dashed lines show the confidence intervals. The blue curves denote the recovery of the protein along the AP-oriented junctions and the pink curves denote the recovery of the protein along the PD-oriented junctions. For each N, 8 ROIs (4 PD and 4 AP) were chosen.

(D-D') FRAP on wild-type (N=11 pupae) and *dachs* mutant wings (N=7 pupae) at 16 hAPF. One-phase exponential NLS fitting of the average normalised intensity for the recovery post-bleaching across pupal wings for Sqh-mKate2 (D) and Ft-EGFP (D'), where error bars represent the standard error of the mean and the dashed lines show the confidence intervals. For each N, 8 ROIs (4 PD and 4 AP) were chosen.

P-values comparing the fluorescence recovery plateaux (y_{max} values) in FRAP experiments were calculated using unpaired t-tests (Table S2.

Figure 3K: ANOVA					
	**Df**	**Sum Sq**	**Mean Sq**	**F value**	**Pr(>F)**
**Condition**	7	0.10278	0.014683	6.197	0.000172
**Residuals**	29	0.06871	0.002369		

Figure 3K: ANOVA with Tukey-Kramer multiple comparisons test							
**Condition 1**	**N**	**Condition 2**	**N**	**Difference**	**Lower**	**Upper**	**Adjusted p-value**
dachs mutant 20-22 hAPF	3	*dachs* 16-18 hAPF	6	-0.10830637	-0.22056842	0.003955683	0.0646768
dpyRNAi 16-18 hAPF	6	*dachs* 16-18 hAPF	6	0.041980763	-0.07473544	0.15869697	0.9359945
dpyRNAi 20-22 hAPF	3	*dachs* 16-18 hAPF	6	0.060983569	-0.081964	0.20393114	0.8586511
dpyRNAi dachs 16-18 hAPF	3	*dachs* 16-18 hAPF	6	-0.064563306	-0.20751088	0.07838426	0.8205814
dpyRNAi dachs 20-22 hAPF	3	*dachs* 16-18 hAPF	6	0.028538449	-0.11440912	0.17148602	0.997806
wild-type 16-18 hAPF	12	*dachs* 16-18 hAPF	6	0.037811103	-0.06326809	0.1388903	0.92246
wild-type 20-22 hAPF	4	*dachs* 16-18 hAPF	6	-0.063103924	-0.1935966	0.06738876	0.7658356
dpyRNAi 16-18 hAPF	6	*dachs* 20-22 hAPF	3	0.1669538	0.02400623	0.30990137	0.0131435
dpyRNAi 20-22 hAPF	3	*dachs* 20-22 hAPF	3	0.185956605	0.02089497	0.35101824	0.0184854
dpyRNAi dachs 16-18 hAPF	3	*dachs* 20-22 hAPF	3	0.06040973	-0.10465191	0.22547137	0.9302761
dpyRNAi dachs 20-22 hAPF	3	*dachs* 20-22 hAPF	3	0.153511485	-0.01155015	0.31857312	0.0833654
wild-type 16-18 hAPF	12	*dachs* 20-22 hAPF	3	0.162784139	0.03229146	0.29327682	0.0067013
wild-type 20-22 hAPF	4	*dachs* 20-22 hAPF	3	0.061869113	-0.09253191	0.21627014	0.8929351
dpyRNAi 20-22 hAPF	3	*dpyRNAi* 16-18 hAPF	6	0.019002805	-0.12394476	0.16195038	0.9998446
dpyRNAi dachs 16-18 hAPF	3	*dpyRNAi* 16-18 hAPF	6	-0.106544069	-0.24949164	0.0364035	0.2688687
dpyRNAi dachs 20-22 hAPF	3	*dpyRNAi* 16-18 hAPF	6	-0.013442314	-0.15638988	0.12950526	0.9999851
wild-type 16-18 hAPF	12	*dpyRNAi* 16-18 hAPF	6	-0.00416966	-0.10524886	0.09690954	1
wild-type 20-22 hAPF	4	*dpyRNAi* 16-18 hAPF	6	-0.105084687	-0.23557737	0.02540799	0.1899055
dpyRNAi dachs 16-18 hAPF	3	*dpyRNAi* 20-22 hAPF	3	-0.125546875	-0.29060851	0.03951476	0.2468977
dpyRNAi dachs 20-22 hAPF	3	*dpyRNAi* 20-22 hAPF	3	-0.03244512	-0.19750676	0.13261652	0.9980108
wild-type 16-18 hAPF	12	*dpyRNAi* 20-22 hAPF	3	-0.023172466	-0.15366515	0.10732022	0.9989594
wild-type 20-22 hAPF	4	*dpyRNAi* 20-22 hAPF	3	-0.124087492	-0.27848852	0.03031353	0.1917853
dpyRNAi dachs 20-22 hAPF	3	*dpyRNAi dachs* 16-18 hAPF	3	0.093101755	-0.07195988	0.25816339	0.6073179
wild-type 16-18 hAPF	12	*dpyRNAi dachs* 16-18 hAPF	3	0.102374409	-0.02811827	0.23286709	0.2150467
wild-type 20-22 hAPF	4	*dpyRNAi dachs* 16-18 hAPF	3	0.001459382	-0.15294164	0.15586041	1
wild-type 16-18 hAPF	12	*dpyRNAi dachs* 20-22 hAPF	3	0.009272654	-0.12122003	0.13976534	0.9999978
wild-type 20-22 hAPF	4	*dpyRNAi dachs* 20-22 hAPF	3	-0.091642372	-0.2460434	0.06275865	0.5463406
wild-type 20-22 hAPF	4	wild-type 16-18 hAPF	12	-0.100915027	-0.21763123	0.01580118	0.1303768

Table S1. Detailed statistical data, related to Figure 3
